# Eating Healthy, Growing Healthy: Impact of a Multi-Strategy Nutrition Education on the Assortments of Beverages Served in Preschools, Poland

**DOI:** 10.3390/ijerph15071355

**Published:** 2018-06-28

**Authors:** Joanna Myszkowska-Ryciak, Anna Harton

**Affiliations:** Department of Dietetics, Faculty of Human Nutrition and Consumer Sciences, Warsaw University of Life Sciences (WULS), 159C Nowoursynowska Str, 02-776 Warsaw, Poland; anna_harton@sggw.pl

**Keywords:** beverages, water, education, child-care menus, inventory reports, preschool, children

## Abstract

Day-care centers are the ideal place for conducting nutrition intervention to improve children’s nutrition. The aim of this study is to evaluate the effectiveness of a multi-strategy childcare-base intervention in improving compliance with nutrition guidelines regarding beverages supply in preschools. The intervention included the staff training, audit, feedback, and ongoing support provided by trained educators. The emphasis was put on adequate nutrition, including recommended beverages, reducing sugar-sweetened beverages and increasing water availability. Enrolled were 478 full-board preschools from Poland (4.2% of all preschools in the country). The assortment of beverages served to children within ten consecutive days was assessed based on menus and inventory reports at the baseline, and three months after the intervention. Education increased significantly the percentage of preschools serving water to meals and between meals (67% vs. 83% and 93% vs. 99%, respectively), fruit/herbal tea (75% vs. 81%), and natural fruit juices (46% vs. 56%). The percentage of preschools offering fruit/soft drinks decreased from 23% to 15%. This study shows that education has a great potential in improving the assortment of beverages served in preschools. Future research is needed to determine barriers in better optimizing the assortment of beverages in preschool settings.

## 1. Introduction

Consuming an adequate amount of fluids is necessary for the proper functioning of the body [1]. For the nutritional value of a diet, the quality of fluids consumed is also important. According to the guidelines [2,3], the most recommended drinks for children are water and nonfat or low-fat milk (or soya/vegetable alternatives), and (optionally) 100% fruit juices in a limited amount. The data show that the assortment of drinks served to children in day care centres (DCCs) is not always in line with the recommendations [4,5]. The majority of the examined DCCs failed to meet the recommendation for proper beverage selection in the preschool menu; three out of four preschools sweetened drinks served to children, and less than 10% offered milk as a beverage [4]. Considering that children in preschools spend up to 10 h a day, and consume from 50 up to 75% of the daily food intake when in care [6,7], the improvement of the quality of served beverages is crucial for improving the quality of their diet. Preschools’ staff have a direct influence on the type of food products purchased and menu offered, therefore education addressed to this group seems to be a promising tool to improve the quality of beverages served and, consequently, the entire menu [8].

Literature exists describing examples of nutrition education programs addressed to child-care facilities [9,10,11,12,13,14,15,16]. Some of them are based on general nutrition and food education provided via media channels (e.g., internet platforms) embedded in the preschool curriculum [7,10,11]. Such programs usually include many institutions, but their disadvantage is the lack of direct contact with the educator (nutritionist/dietician), lack of personalized approach in recommendations, and feedback for participating institutions. In some cases, the effectiveness of educational activities is not verified or the verification is based only on survey methods. In Poland, an example of such a program is the educational campaign “Mom, Dad, I prefer water” addressed to parents and kindergartens [13]. As part of the program, participating kindergartens gain access to educational materials concerning the importance of water in a healthy diet, available on the internet platform (ready-to-use classroom materials for children). Kindergartens can also participate in thematic competitions, and after completing a certain number of activities, they can apply for a prize. In year 2018, the prize for the 5 kindergartens actively involved in promoting water consumption is the interactive floor “Magic Carpet” and themed gifts for all children [11]. However, despite the fact that this program has been implemented since 2009, and more than 1000 DCCs have been enrolled, there is no information available on its influence on increasing the availability/consumption of water by children, as evaluation of effectiveness is not planned within the program. In other programs direct education methods (i.e., counselling and/or workshop sessions for staff and/or children and/or parents) provided by a qualified nutrition specialist were used [11,14,15]. The advantage of this form of education is a possible individual approach, while usually they are available for limited numbers of institutions. In Poland, Kozioł-Kozakowska et al. [14] carried out an educational program aimed at increasing the consumption of fruit and vegetables by pre-school children. Within the program, enrolled children participated in the workshops on balanced nutrition, and the role of vegetables and fruits in the diet, while parents had the opportunity to participate (free of charge) in lectures on the same subject. About 250 children from 6 kindergartens in Cracow were covered by direct education, however no data on program effectiveness is available. 

Although food education programs continue to increase in number, there are smaller numbers of studies that evaluate the real impact (i.e., improvement of children’s nutrition and/or improvement of menus offered to children) of these programs, especially in DCCs settings [11,15,16]. It should be emphasized that the effectiveness of educational programs should be performed with objective methods taking into account the actual change in nutritional practices in the DCC. Some authors as the evaluation of the effectiveness of educational programs take only the increase in knowledge of the personnel participating in the training [15]. However, the associations between higher nutrition knowledge and nutritional behaviors and practices are weak [17]. Therefore, in the case of education addressed to DCCs, as an objective method of effectiveness assessment, the change in the menu rather than nutritional knowledge of staff should be used. Moreover, the assessment should be based on analyses of the inventory reports or observation in DCCs. There is evidence that menus (especially when prepared in advance) or information received during the interview may not reflect food products served to children [18]. 

This study aimed to evaluate the effectiveness of a multi-strategy childcare-base intervention in improving compliance with nutrition guidelines regarding beverages and water supply in preschools in Poland.

## 2. Materials and Methods

### 2.1. General Information

The presented research is part of an educational and research program *Eating healthy*, *growing healthy* aimed at nurseries and preschools in Poland in 2015–2017. The main goal of the project was to improve the quality of nutrition in DCCs by increasing the knowledge and dietary awareness of personnel in participating institutions. The educational framework was designed by an expert advisory group in health, nutrition and development of children, consisted of dietitians, behavioral scientists, implementation scientists and a health care practitioner (pediatrician). The curriculum included lectures/workshops on: General recommendations of balanced nutrition; Water and its role in children diet; Sugar in children diet; Prevention of food allergies; Fussy eater or food neophobic; Strong bones and teeth—the role of vitamin D and calcium; Salt in children diet; Attractive presentation of the meals; Plump, overweight, obese—how to recognize the problem; Limits of child’s choice; Involving kids in cooking; Servings size—self-eating, self-deciding. Thematic scope of the program was selected based on a theoretical framework of education of preschool age children in Poland [19], and previous research evidence on nutrition related practices in DCCs (observed irregularities in the implementation of nutrition recommendation in care and educational centers) [20,21]. The program offered the institutions the possibility of direct and indirect participation. In the case of an indirect participation, the institutions obtained access to a special website platform, on which all educational materials were available without any charge. In addition, these DCCs received monthly newsletters on different nutritional topics. However, these institutions did not have the direct contact with the educator, nor did they receive personalized recommendations, nutritional analyses of their menus, and/or feedbacks. In the case of direct participation in the program, staff education was conducted by specially trained educators (dieticians and nutritionists). Educational program included lectures, workshops and counselling/consultation session (a total of 24 h for all personnel involved in ordering food products, planning and preparing menus in each institution). The effectiveness of education was assessed through the analysis of nutrition quality (frequency of serving selected products/beverages, and energy and nutrients content of the menus) in direct participating institutions conducted before (at baseline) and about 3 months after the training (post baseline). Participating institutions received verbal and written feedback after the first and second audit. Participation in the program was free of charge for all DCCs. Detailed information about the project *Eating healthy*, *growing healthy* are available on website [22], and in the previously published article [4]. Nurseries and preschools were recruited to the program through written invitations sent directly to the institutions (a list of institutions from all over the country was obtained from relevant municipal offices and education departments), and through media channels addressed to these institutions (e.g., websites, journals, periodicals). In total, 2638 institutions participated in the program, of which 1347 were directly involved. Direct education covered 13,214 employees of these institutions. In the present study, we focused only on direct participating preschools as only these DCCs were included in a detailed assessment of nutritional practices.

### 2.2. Study Setting

For the present study, the preschools with the completed nutritional education program were selected, and only these offering full board, i.e., 2 main meals (breakfast, lunch) and 1 or 2 snacks. The analysis excluded childcare facilities working in the part-time system due to a lower number of meals and beverages offered. The other criterion of exclusion was the lack of full nutritional documentation provided for the first and/or the second analysis. The above criteria were fulfilled by 478 preschools, and data from all these institutions were analyzed in this paper.

Institutional Review Board approval was not necessary for this project because human subjects were not involved, as per University of Life Sciences Center Institutional Review Board guidelines. No personal data were collected concerning preschools’ staff and children within the project. Preschools were informed about the purpose and scope of the program, and the possibility of withdrawing from it at any stage without giving any reason.

### 2.3. Analyses of the Asortment of Beverages Served before and after Education

This analysis focused on the types of beverages served with meals and water availability between meals before and 3 months after nutrition education. Data regarding the assortment of beverages offered in preschools were obtained by face-to-face interview during the visit in all examined preschools. We collected information on the typical beverages offered in preschool, free access to water between meals, sweetening practices (whether beverages were sweetened and what type of sweetener was used), as well as information characterizing the institution (including type of DCCs, model of nutrition organization, number of children, financial rate per child per day). Then, data were verified on the basis of 10-day menus and inventory reports (10 consecutive days). Based on these documents, we checked whether the beverages declared by the preschool during the interview were present at least once during 10 consecutive days, and only these beverages were included to the further analyses. In the previous article, we analyzed the assortment of beverages offered to children in preschools in Poland at baseline (before education) [4]. In 720 examined preschools, children were served: cocoa and milk coffee substitute, compote, tea (black, green), herbal/fruit tea, pure water, juices 100% natural, fruit/soft drinks (not 100% juices), milk, water with honey or fruit syrups, fruit-milk shakes, milk tea (black), water with fresh lemon/orange/mint, fermented milk beverages (kefir, yoghurt), liquid jelly/fruit starch jelly, water available between meals. The detail characteristic and nutritional value of served beverages is available elsewhere [4]. Due to statistical reasons in the present paper, only typically served beverages (offered in at least 10% of preschools) were selected to assess the impact of education. The above criteria were fulfilled by 7 types of beverages: water, tea (regular/black), herbal/fruit tea, compote (beverage made of typical Polish fruits as apples, strawberries, cherries, currants boiled in large amount of water, typically sweetened with sugar), 100% juice, soft/fruit drink and cocoa/milk coffee substitute (usually wheat; prepared with milk instead of water, typically sweetened with sugar). In the case of water, we checked whether it was served to meals and freely available for children between meals. 

### 2.4. Statistical Analysis

Statistical data processing was performed using Statistica version 12 (Copyright©StatSoft, Inc., 1984–2014, StatSoft Polska Sp. z o.o. manufacturers, Cracow, Poland). For the general characteristic of preschools participating in the study: number of children, the amount of money allocated per day to feed a child (financial rate) means, standard deviations (SD), and medians were calculated. Data on beverage supply were analyzed in total group and by the type of facility (public vs. non-public) before and after education. Statistical significances for qualitative variables were determined using Pearson’s chi-square test. Additionally, contingency coefficient Cramér’s V was used to indicate the strength of association between categorical variables. In the case of quantitative variables, the Shapiro-Wilk statistic for testing normality was used. Due to the lack of normal distribution of quantitate data, U Mann–Whitney test was used to check the significant differences. The differences were considered significant at *p* ≤ 0.05.

## 3. Results

The study involved 478 preschools from the total number of 11,331 institutions in Poland [23] (4.2% of all preschools in the country). The majority (74%) were public preschools, characterized by a higher average number of children, a lower amount of money allocated per day to feed one child, and mainly maintaining own kitchen. To compare the size of preschools, lower and upper quartile due to the number of children attending was calculated (Q1 = 68, Q3 = 143). Medium-sized institutions dominated in public preschools, while in non-public institutions—small ones. The detail characteristic of participating institutions is presented in Table 1. In total, 53,194 children aged 3–6 attended in all participating institutions in the analyzed period. 

Table 2 presents the types of beverages served in the preschools, overall, and by public and non-public preschools, before and after education. In the total group, education significantly increased the percentage of preschools serving fruit/herbal tea, natural fruit juices and water to meals and between meals. At the same time, the number of institutions offering soft/fruit drinks has decreased significantly. When comparing the effectiveness of education in the public and non-public preschools, some differences were observed. In the non-public institutions, education proved to be an effective tool only in the case of water served as a beverage with meals. However, in the case of all significant differences, the value of the Cramer coefficient indicates a low level of dependence between variables.

## 4. Discussion

In Poland, 84% of children in the age group of 3–5 years were enrolled in different types of DCCs in 2015–2016 [23]. Considering the number of children, and the time spent in care facilities, DCCs largely determine the dietary intake of children, but they are also an ideal place for shaping the correct eating habits. In addition, children often continue their early diet habits into adulthood [24]. In Poland there is only a very general legal regulation regarding nutrition in kindergartens [25]. The only requirement for beverages concerns the limitation of the addition of sugar to beverages prepared at the DCCs from scratch to 10 g per 250 mL (8.5 oz.) of the served beverage. However, there are no restrictions regarding the quality and quantity of other beverages, including those purchased as ready for consumption. There are also evidences, that the existing legal regulations are not always respected by the institutions [26]. Considering the above, educating the preschools staff in the field of balanced nutrition is crucial for increasing the awareness of the role of the correct diet and improving nutrition in DCCs.

Beverages may contribute to the quality of children’s diets. However, in terms of the healthy diet, in addition to the quantity, the quality of the beverages is very important [1]. The best choice is water [27], however, for children a beneficial alternative to water can be milk as a source of calcium and vitamin D_3_ [28]. Water can be served during meals and snacks along with milk and other healthier beverages [29]. In our study, education caused a significant increase in number of preschools serving water to a meal, both in public and non-public public institutions. In the case of access to water between meals, education has benefited significantly only public institutions. However, it is worth emphasizing that a majority of the institutions (but more non-public) provided children with free access to water before the educational program. Considering that water is a low cost, calorie-free drink, there is still some room for improvement, especially in case of availability of water during the meals. Increasing drinking water access in preschools is a step in the right direction toward encouraging children’s water intake. By increasing the water intake instead of sugar-containing beverages, the calorie content of children’s diets may be reduced [30].

Natural fruit juices can supplement children’s diet with vitamins and minerals, in the case of juices extruded with pulp, also in dietary fiber [31,32]. However, fruit juices also contain significant amounts of simple sugars, mainly fructose, so their amount should be limited to a maximum of 1 serving (4–6 ounces/day) per day [33]. In our education program, we emphasized that fruit juices (in the amount not exceeding 1 serving) can be an element of a properly balanced diet, but they do not have any advantage over fresh fruits. Before education, fruit juices were served in less than half of examined institutions. They were served more often by public preschools, which was unexpected because these institutions have a lower budget, and natural fruit juices are a rather expensive food product. After education, fruit juices were served in a higher number of DCCs, but statistical significance concerned only public institutions. It seems that a lower budget is not an obstacle to the purchase of more expensive products. 

The research results alert that fruit/soft drinks (sugar sweetened beverages) have a growing share in the beverages consumed by children recently [34]. They are eagerly chosen by children because of the sweet taste and attractive colors, but also the advertising influences children/caretakers preferences and purchase requests [35]. These types of beverages are especially inadvisable to children due to the high proportion of sugar and low nutritional value [6]. It is worth noting that these types of drinks were served in less than every fifth DCC. Education significantly reduced the number of institutions serving soft and fruit drinks; however, this favorable change concerned only public facilities. Since still more than every tenth institution usually gives children this type of drink, it seems advisable to continue education in this area. Both juices and fruit drinks are a source of free sugars. However, considering their nutritional value (vitamins, dietary fiber), reducing the frequency of serving fruit/soft drinks in favor of juices seems a beneficial effect of education.

Tea (regular, black) is also not recommended for young children. The phenolic and tannin compounds present in the tea leaves may limit the absorption of iron (specially non heme iron), which in the case of low content of this mineral in the diet may increase the risk of anemia in children [36,37]. A more recommended choice might be fruit or herbal infusions not containing phenolic and tannin compounds, but only if they are served unsweetened. Black tea is commonly consumed in Poland, which also translates into menus in preschools. Education did not affect the number of institutions providing tea to children, but it increased the share of preschools serving fruit tea. This change was observed specially in public institutions, in which this beverage appeared less frequently before education. These are favorable trends, but still require further action. An additional argument in favor of limiting the administration of tea to children is its usual sweetening with sugar [2]. Due to the recipe, herbal or fruit teas are sweetened less often due to their naturally sweet taste.

While not endorsing cocoa and milk coffee substitute over milk, these beverages prepared without added sugar may contribute to calcium and vitamin D intake. In Recommendations for Healthier Beverages (2013), flavored milk was admitted due to its popularity among children; however, a calorie limit of no more than 130 calories per 8-ounce serving was set to limit the amount of added sugar [38]. In Poland, cocoa and milk coffee substitute are very popular in DCCs, unlike milk, which as a beverage was served by less than 10% of preschools [2]. In our education, we focused on promoting milk without any additives as a recommended drink, but this did not cause any changes in the DCCs, both public and non-public. Additional research should be conducted on the causes of this situation. 

In a majority of preschools, children received compote to drink. Compote, due to the main ingredient (fruit), contains mainly simple sugars and some amounts of vitamin C. But during thermal processing (boiling), more than 50% of vitamin C is missing, so compote, opposite to fresh fruit or natural 100% fruit juices, is not a good source of it [39]. The education emphasized the advantage of offering children fresh fruits over serving them in processed form. Compote and fruit juices are a source of free sugars. World Health Organization (WHO) recommends limiting consumption of free sugars to a maximum of 10% of energy from the diet. Moreover, WHO suggested that halving this limit to 5% could have additional health benefits [40], which in the case of a child in pre-school age is not more than 1 serving of processed fruits (compote or juice). Education was not efficient in decreasing the number of DCCs offering compote to children. It can be hypothesized that the compote is perceived by personnel as a “healthy” product, despite the addition of sugar. Due to its popularity, it would be recommended to examine what factors inhibit the recommended changes.

Our research shows the effectiveness of education in changing the assortment of served beverages in preschools. They also underline some differences in the effectiveness of education in public and non-public institutions. It can be concluded that a lower budget and/or a larger number of children is not an obstacle to introducing beneficial changes in a relatively short time.

Our research has some limitations. Although the study covered a large number of institutions (public and non-public) from all over the country, they are not representative of all DCCs in Poland due to inclusion methods (DCCs enrolled to the program). The introduced changes were assessed only once, about 3 months after intervention. In order to assess the long-term effect of program, it would be beneficial to repeat the assessment after a longer time (e.g., 1 year).

## 5. Conclusions

This study shows that direct, intensive nutrition education has a potential in improving the assortment of beverages served in preschools. It also indicates the differences in the effectiveness of education in case of different beverages, and different type of institution (public vs. non-public). It can be assumed that the change in the case of beverages commonly served and preferred by children due to sweet taste is more difficult for institutions. Future research is needed to determine barriers in better optimizing (e.g., increasing milk servings) the assortment of beverages in preschools.

## Policy Implications

The results of the research can be used in planning other educational programs addressed to DCCs. They can also be used to create practical recommendations regarding the supply of beverages in pre-school institutions in Poland.

## Figures and Tables

**Table 1:** General characteristics of preschools participating in nutritional education (*n* = 478).

Variable	Public (*n* = 355)	Non-Public (*n* = 123)	Total (*n* = 478)
Number of children:			
Mean ± SD	123 ± 56 *	77 ± 63	112 ± 58
Median/Min/Max	123/9/411	55/10/415	110/9/420
Financial rate per child/day/PLN ^1^:			
Mean ± SD	5.8 ± 1.4 *	8.2 ± 2.1	6.4 ± 1.9
Median/Min/Max	5.5/3.0/15.0	8.0/4.5/15.0	6.0/3.0/15.0
Model of nutrition organization: (number of preschools/%) **			
Kitchen	337/95%	63/51%	400/84%
Catering (external provider)	12/3%	44/36%	56/12%
Internal catering	4/1%	6/5%	10/2%
Mixed	2/0.5%	10/8%	12/3%
Size category of preschool: (number of preschools/%) **			
Large	102/29%	15/12%	117/24%
Medium	205/58%	34/28%	239/50%
Small	48/14%	74/60%	122/26%

Notes: ^1^ 1 PLN (Polish zloty) = ~0.24 EUR; * significant differences between public and non-public preschools, U Mann-Whitney test *p* < 0.05; ** significant differences between public and non-public preschools, chi ^2^ Pearson test *p* < 0.05; Percentages may not add up to 100 due to rounding.

**Table 2:** Assortment of beverages offered to children in preschools before and 3 months after education: public (*n* = 355), non-public (*n* = 123), and data for the total group (*n* = 478).

Beverages Offered in Preschools	Before Education (Number of Preschools/%)	After Education (Number of Preschools/%)	*p*-Value before vs. After Education/Cramér’s V
Water:			
Public	241/68%	298/83%	*p* < 0.001 */V = 0.18
Non-public	79/64%	101/82%	*p* = 0.00 */V = 0.19
Total	320/67%	399/83%	*p* < 0.001 */V = 0.18
Water available between meals:			
Public	328/92%	352/99%	*p* < 0.001 */V = 0.16
Non-public	115/94%	121/98%	*p* = 0.06/V = 0.12
Total	443/93%	473/99%	*p* < 0.001 */V = 0.15
Juice (100% natural):			
Public	176/50%	213/60%	*p* = 0.00 */V = 0.10
Non-public	45/37%	53/43%	*p* = 0.29/V = 0.07
Total	221/46%	266/56%	*p* = 0 .00 */V = 0.09
Soft/fruit drink:			
Public	83/23%	51/14%	*p* = 0.00 */V = 0.11
Non-public	26/21%	21/17%	*p* = 0.42/V = 0.05
Total	109/23%	72/15%	*p* = 0.00 */V = 0.10
Tea (regular/black):			
Public	297/84%	290/82%	*p* = 0.49/V = 0.03
Non-public	99/80%	92/75%	*p* = 0.28/V = 0.07
Total	396/83%	382/80%	*p* = 0.24/V = 0.04
Herbal/fruit tea:			
Public	254/72%	281/79%	*p* = 0.02 */V = 0.09
Non-public	105/85%	105/85%	*p* = 1.00/V = 0.00
Total	359/75%	386/81%	*p* = 0.03 */V = 0.07
Cocoa and milk coffee substitute:			
Public	345/97%	346/97%	*p* = 0.82/V = 0.01
Non-public	102/83%	107/87%	*p* = 0.37/V = 0.06
Total	447/94%	453/95%	*p* = 0.41/V = 0.03
Compote:			
Public	331/93%	332/93%	*p* = 0.88/V = 0.01
Non-public	102/83%	104/85%	*p* = 0.73/V = 0.02
Total	433/91%	436/91%	*p* = 0.73/V = 0.01

Notes: * significant differences between, chi ^2^ Pearson test *p* < 0.05; Percentages may not add up to 100 due to rounding.
